# Medical Press

**Published:** 1849-12

**Authors:** 


*Medical Press.*—In Paris there are 24 medical journals, of which 3 are published quarterly, 17 monthly, 2 weekly, and 2 three times a week. Some few of the provincial medical societies publish bulletins, and a medical journal is published at Bordeaux, Lyons, Marseilles, Montpellier, and Strasburg.—*Roubaud's Annuaire Medical,* 1849.

